# Impact of COVID-19 on people living with HIV: A review

**DOI:** 10.1016/j.jve.2020.100019

**Published:** 2020-10-15

**Authors:** Sandeep Prabhu, Selvamuthu Poongulali, Nagalingeswaran Kumarasamy

**Affiliations:** a University of Washington, Seattle, USA; b VHS-Infectious Diseases Medical Centre, Voluntary Health Services, Chennai, India

**Keywords:** COVID-19, HIV, Epidemiology, Pathogenesis

## Abstract

There is great concern about the impact of COVID-19 among the nearly 40 million people living with HIV (PLWH) worldwide. In this review, we surveyed current literature and found no evidence of higher prevalence of COVID-19 among PLWH but equivocal data on increased mortality and worse clinical outcomes. Having HIV does not confer protection against severe manifestations of COVID-19. Several studies looking at antiretroviral drugs against HIV to treat SARS-CoV-2 have shown no mortality benefit. Thus, there is no indication to change antiretroviral therapy (ART) regimens among virologically suppressed PLWH to prevent COVID-19. HIV care delivery has been adversely impacted in several countries during this pandemic but has created an opportunity for accelerating effective strategies like multi-month ART. Decentralizing HIV care in low-resource settings and incorporating telemedicine in high-resource settings will be critical in mitigating shocks to healthcare systems in the future.

## Introduction

As of August 26, 2020, over 24 million people worldwide have been diagnosed with COVID-19, the disease caused by the novel coronavirus (i.e., SARS-CoV-2). Of the nearly 40 million people living with HIV (PLWH) worldwide, most live in countries with poor healthcare infrastructure and the majority are over age 50 in several countries. Advanced age and coexisting chronic diseases, such as hypertension, diabetes, and obesity are associated with severe manifestations and higher mortality from COVID-19, putting many PLWH at increased risk.^1^ Alarmingly, two of the three countries with the highest number of HIV infections- South Africa and India-are also among the countries with the highest COVID-19 burdens.^2^

## Methods

For this scoping review, we searched PubMed for original research, reviews, and viewpoint articles on HIV and COVID-19 using the terms “HIV”, “human immunodeficiency virus”, “AIDS”, or “acquired immunodeficiency syndrome” for HIV and “COVID”, “COVID-19”, “Sars-CoV-2”, or “novel coronavirus” for COVID-19. 349 articles published in English from Jan 1, 2019 to Jul 31, 2020 were reviewed. Papers were retained for this review if they discussed the epidemiology, pathogenesis, pharmacology, or access to care among individuals with HIV and COVID-19 coinfection. We also reviewed reports from the WHO, CDC, and European AIDS agencies, abstracts from the AIDS 2020 conference, and press reports related to relevant articles. A scoping review was chosen over a systematic review to enable us to incorporate a variety of study designs given the paucity of randomized controlled trials currently. Pre-prints were included when we believed the data presented were potentially ground-breaking in an area that is evolving rapidly.

## Results

### Epidemiology of COVID-19 in PLWH

Observational and case control studies looking at the prevalence, clinical course, and mortality of COVID-19 among PLWH are being increasingly published, whose key takeaway points we summarize below.

### Prevalence estimates

Current estimates of the prevalence of HIV and COVID-19 coinfections come from observational studies in several countries. Vizcarra et al. noted that 51 individuals in their Madrid-based cohort of 1339 PLWH contracted COVID-19, giving a prevalence of 3.8%.^3^ Richardson et al. calculated a 0.8% prevalence of HIV among 5700 patients admitted with COVID-19 in 12 New York area hospitals.^4^ Both studies are likely underestimates because they exclude outpatients and the Madrid study noted restrictive testing criteria. In the Veterans Aging Cohort Study consisting of 30,891 PLWH and 76,745 HIV-negative individuals, the COVID-19 positivity rate in both groups were quite similar (9.7% vs. 10.1%) at the end of June 2020.^5^ Taken together, there is no evidence that COVID-19 prevalence among PLWH is significantly different from that in the general population, an observation acknowledged by multiple European national HIV/AIDS organizations.^6^

Multiple studies have noted that PLWH with COVID-19 have a median age about a decade lower than individuals without HIV, despite a similar prevalence of comorbidities.^3^^,^^7^^,^^8^ This is likely related to the fact that biological age is advanced in PLWH by several years, which raises the possibility that the age threshold for determining high-risk among PLWH may differ from that in the general population, which could have marked public health implications.^9^

Ethnic disparities in COVID-19 incidence that have been noted in the general population are unfortunately seen among PLWH as well. Black individuals in a cohort of HIV-positive individuals at a single hospital in London had a significantly higher odds ratio of being hospitalized with COVID-19 than non-Black individuals (OR 12.2; 95% CI [1.62, 92]).^10^ In the Veterans Aging Cohort Study (VACS), Black and Latino veterans were 70% and 40% more likely than Caucasians to contract COVID-19, independent of HIV status, respectively. The reasons for this are multifactorial and include a higher prevalence of comorbidities, increased occupational exposure, and other socioeconomic factors, which have been well described elsewhere.^11^

### Mortality estimates

Several small studies have noted that COVID-19 mortality among PLWH does not differ from the general population. A case-control study in New York City that compared 88 PLWH with COVID-19 and matched them to individuals without HIV-1 infection on age, sex, race/ethnicity, and week of infection found no difference in need for mechanical ventilation or mortality.^12^ No difference in hospitalization, ICU admission, intubation, or death by HIV status was noted in the VACS.^5^ Similarly, Lee, et al. found no mortality difference by HIV status and even noted that individuals with HIV were more likely to show clinical improvement/be discharged within 28 days of admission.^13^

Evidence of significantly increased COVID-19 mortality risk among PLWH comes from a province-wide cohort of individuals accessing public healthcare in Western Cape, South Africa and two studies using national databases in the United Kingdom.^8^^,^^14^^,^^15^ In the Western Cape study, HIV-positive individuals have a significantly higher hazard ratio of mortality than individuals without HIV after controlling for age, diabetes, hypertension, and chronic kidney disease (HR 2.75; 95% CI [2.09, 3.61]).^15^ Even without controlling for obesity, tobacco use, and socioeconomic status, this first report from a high HIV and tuberculosis (TB) prevalence setting is highly concerning, especially since active and past TB was independently associated with increased mortality in this study. Concerningly, nearly half of the PLWH in the same sample currently have or have had TB. While these results from the Western Cape are from a pre-print and not yet peer reviewed, they are compelling due to the magnitude of effect seen as well as the large sample size of individuals. However, the external validity of these findings in settings where HIV and TB are far less common is unclear.

The data from the ISARIC database, consisting of 53,992 individuals with COVID-19, found that hospitalized PLWH with COVID-19 had a 63% higher mortality than their HIV negative counterparts, after adjusting for age, ethnicity, comorbidities, and disease severity when they presented to the hospital.^8^ Similarly, results from the OpenSAFELY dataset, consisting of clinical data from hundreds of primary care practices through the UK, found a more than two-fold increase in mortality among PLWH with COVID-19 compared to those without HIV-1 infection (HR 2.30; 95% CI [1.55, 3.41]) after adjusting for age, ethnicity, and several comorbidities.^14^ Notably, both studies lack data on HIV-related parameters such as antiretroviral therapy (ART) use, HIV-1 viral load, CD4 T cell count, and prior opportunistic infections, all of which are important confounders. While both studies note that over 90% of individuals diagnosed with HIV-1 receive ART, it is impossible to know if the small number of PLWH constitutes a representative sample case-series in the UK. Consequently, we interpret this data with caution.

### Comorbidities

The correlation between COVID-19 mortality among PLWH and other comorbidities is noted in multiple studies. Vizcarra et al. noted a higher prevalence of comorbidities among PLWH with COVID-19 than in individuals without HIV (63% vs. 38%; p ​< ​0.01).^3^ 7 of 9 PLWH with COVID-19 died in a case series from the Bronx; none of the individuals had >50 copies of HIV-1 RNA, but everyone aged >50 in this case series died.^16^ Are comorbidities among PLWH the drivers of poor outcomes from COVID-19 rather than HIV itself? Questions about whether comorbidities are confounders or effect measure modifiers are important and still unclear.^17^^,^^18^

Larger prospective studies are required to elucidate this further and assess if a synergistic effect between comorbidities and HIV exists. Currently published studies mostly feature individuals with undetectable HIV-1 viral loads, making it challenging to generalize to those with poorly controlled HIV.^7^^,^^10^^,^^19^^,^^20^ We call for more work on broader samples of PLWH (including those with advanced disease).

### Pathogenesis

Do PLWH with COVID-19 have worse outcomes than the general population? There are two contrasting hypotheses for this question. The first one, from Mascolo and colleagues, hypothesizes that relative immunosuppression from HIV may make PLWH more susceptible to contracting COVID-19, but lymphopenia may protect against severe manifestations of COVID-19 due to an inability to mount an overactive T cell response.^21^ The other one draws from prior studies showing that HIV-1 infection is characterized by a chronically elevated inflammatory state, which when combined with the cytokine storm seen in severe COVID-19, should theoretically worsen outcomes.^22^ Two case-series and several observational studies provide further insights.

Vizcarra et al. found in their case-series that individuals who became critically ill tended to have low nadir CD4 T cell counts (<200 ​cells/μL).^3^ Härter et al. described 32 patients with HIV-1 and COVID-19, of whom only two required mechanical ventilation with both having detectable HIV-1 viral loads.^19^ Finally, Ho, et al. performed a retrospective analysis of 72 individuals with HIV-1 and COVID-19 in New York and found that individuals who died had lower nadir lymphocyte counts and higher levels of inflammatory markers (C-reactive protein, IL-6, and IL-8) than those who survived.^23^ In contrast, Patel, et al. noted a higher rate of intubation and death among PLWH who were virally suppressed compared to those who were not and an increased likelihood of intubation among PLWH with higher CD4 T cell counts.^24^ Lee et al. found that PLWH in one London hospital were discharged more quickly than their matched HIV-1 negative counterparts.^13^

Clearly, the evidence is equivocal. The Vizcarra et al. and Härter et al. studies lack control groups, and have small sample sizes. The Patel et al. study does not control for the COVID-19 treatment patients received, which is a critical variable.^24^ Summarily, there is no compelling evidence for better or worse clinical outcomes among PLWH with COVID-19 compared to their HIV-1 negative counterparts; further work is needed to clarify this issue better.

### Is COVID-19 an opportunistic infection?

Most studies of PLWH with COVID-19 published thus far include those with well-controlled HIV-1, suggesting that COVID-19 is not an opportunistic infection (OI). Furthermore, no reports of immune reconstitution inflammatory syndrome (IRIS) among PLWH with COVID-19 have been published, so ART initiation should not be delayed in individuals with COVID-19 who are newly diagnosed with HIV-1. In this era of heightened COVID vigilance, we would like to underscore the importance of thoroughly evaluating respiratory complaints among PLWH, so that OIs (e.g., *Pneumocystis jirovecii* pneumonia [PJP], atypical [TB]) that resemble COVID-19 both clinically and radiographically are not missed.^25^^,^^26^ Conversely, it is important that atypical clinical presentations of HIV-1 remain on the differential diagnosis and the individuals are offered HIV-1 testing when appropriate.

### Development of antibodies to SARS-CoV-2

It remains a matter of debate how protective antibodies against SARS-CoV-2 are, how long they last, and if they help prevent reinfections. These questions not withstanding a single case-report suggests a slower rate of protective antibody development against SARS-CoV-2 among individuals with HIV-1 compared to those without. While a case-series from Chongqing of hospitalized patients with COVID-19 (HIV-1 status unknown) reported a median time from start of symptoms to IgG seroconversion of 13 days, a case-report of a PLWH noted that it took several weeks for a patient to develop an IgM antibody response against SARS-CoV-2.^25^^,^^27^ Larger scale studies are needed to confirm if this pattern is confirmed. Furthermore, we do not know if waning antibody titers will still prevent COVID-19 reinfection due to an amnestic response. Until such data is available, guidelines on self-isolation after COVID-19 diagnosis used for the general population should be followed for PLWH.

PLWH admitted with COVID-19 must continue to receive ART and appropriate OI prophylaxis if CD4 T cell counts decrease. Furthermore, when therapeutics targeting COVID-19 are initiated, clinicians must be cognizant of drug-drug interactions with ART to prevent adverse effects.

There is still much to be gleaned on the natural history of COVID-19 infection in PLWH, with many prospective observational studies underway.^28^ Unanswered questions include how the different complications of each infection manifest in HIV-1 and COVID-19 coinfection. For example, hospitalized patients with COVID-19 have a high incidence of venous thromboembolism.^29^ PLWH have an elevated inflammatory state, especially with advanced/uncontrolled disease.^22^ Whether this results in drastically adverse outcomes is still unknown. Upcoming studies should incorporate a wide array of PLWH, including those with advanced HIV/low CD4 T cell counts.

### Pharmacology

The search for pharmacologic therapies against SARS-CoV-2 has initially largely focused on studying existing drugs targeting other viruses, including antiretroviral agents (ARVs) used to treat HIV. Initial genomic analysis of SARS-CoV-2 has indicated that viral enzymes are similar in sequence to the respective enzymes in SARS and MERS.^30^

Early optimism about protease inhibitors such as lopinavir/ritonavir (LPV/r) arose from early guidance from Wuhan and prior data suggesting efficacy against SARS and MERS.^31^ As seen in Table 1, the three randomized trials looking at outcomes from LPV/r use in COVID-19 provide no evidence that it improves clinical outcomes among hospitalized patients with COVID-19 and may even lead to clinical progression.

**Table 1:** 

Study	Study design	Location (sample size)	Inclusion criteria	Key exclusion criteria	Intervention	Key outcomes	Results
Cao et al.^36^	Randomized, open label trial	One hospital in Wuhan, China (n ​= ​199)	Hospitalized patients who were hypoxemic on room air or had an arterial pO_2_/fraction of inspired oxygen ratio<300	Individuals with HIV	LPV/r ​+ ​standard of care (SOC) vs. SOC x 14 days	Time to clinical improvement, mortality difference	No difference in time to clinical improvement among those with LPV/r compared to SOC (HR 1.24; 95% CI [0.9,1.72]) and mortality (−5% difference; 95% CI [-17.3, 5.7])
Li et al.^37^	Double blinded RCT	One hospital in Guangzhou, China (n ​= ​86)	Hospitalized patients aged 18–80 with PCR confirmed SARS-CoV-2 infection -Mild clinical status (see exclusion criteria)	Individuals with clinical or radiologic signs of pneumonia	LPV/r vs. arbidol vs. no antiviral therapy	Time to COVID-19 seroconversion from (+) to (−)	No difference in time to seroconversion (9.0 vs. 9.1 vs. 9.3 days); LPV/r group more likely to progress from mild/moderate to severe/critical
Solidarity trial^38^	Double blinded RCT	Multinational (n ​= ​1000)	Age≥18, hospitalized patients		Hydroxychloroquine ​+ ​SOC, LPV/r ​+ ​SOC, remdesivir ​+ ​SOC, SOC		LPV/r arm discontinued on July 4, 2020 due to lack of mortality reduction

Nucleoside reverse transcriptase inhibitors (NRTIs), which target block viral RNA synthesis by acting on the RNA-dependent RNA polymerase, form the backbone of first-line ART against HIV-1 and are being actively studied against SARS-CoV-2. Del Amo et al. carried out a cohort study in which they note a lower risk of contracting COVID-19 among individuals on TDF/FTC compared to other regimens and fewer complications.^32^ The investigator is leading the EPICOS trial in Spain looking at whether tenofovir, either in conjunction with emtricitabine or both emtricitabine and hydroxychloroquine, can prevent COVID-19 among healthcare workers.^33^ We look forward to seeing the results of this trial noting that the current evidence for NRTIs as treatment for COVID-19 is not proven.

In summary, there is currently no indication to change ART regimens among virologically suppressed PLWH to prevent COVID-19. Until new data from ongoing trials emerges, national governments must work to ensure that medications like LPV/r, a second line of treatment for some PLWH, are maintained and not diverted.

### Vaccines

We are concerned that PLWH are being excluded from large phase III studies assessing the efficacy of a SARS-CoV-2 vaccine.^34^ Considering the evidence that current ART regimens have no protective effect against COVID-19, we do not see a clinical justification for excluding people with well-controlled HIV-1 when other types of patients with stable medical conditions remain eligible to participate in these studies. This unconscionable exclusion can further reduce access to PLWH, as occurred with Zostavax several years ago, when initial studies on its efficacy excluded virally-suppressed PLWH, who did not obtain insurance coverage until a separate efficacy trial among PLWH was performed.^35^

### The pandemic’s impacts on HIV care delivery

Years of progress in HIV testing and treatment are in jeopardy due to the COVID-19 pandemic, which has created unprecedented challenges to HIV care delivery worldwide.

### Individual level challenges

The fear of contracting COVID-19 has led to decreased engagement with care among PLWH in several countries.^39^, ^40^, ^41^, ^42^ The reasons for this are unsurprising. People who have faced stigma for decades due to their HIV status and live under the burden of a chronic virus are hesitant to engage in care when the prospect of being infected with another, more deadly virus is real. This fear has even led some to question the safety of going to pharmacies to collect ART.^41^

National lockdowns have also significantly hindered access to care. In Uganda, nationwide public and private transportation suspensions have made distant clinics all but impossible to access for many.^40^ Migrant workers in cities have retreated to the rural areas they are from, likely losing access to HIV care in the cities where they lived.^42^ Even when people can access clinics, the financial stress from loss of livelihoods make it harder for them to pay for care.

Finally, physical distancing guidelines can exacerbate the high levels of isolation that older individuals with HIV already face.^43^ Despite no definitive evidence that PLWH are at higher risk of contracting COVID-19, individuals may fear that they are and result in them presenting late to care. Depression is the second most common mental health disorder (after substance use) among PLWH and constitutes an additional barrier to care.^44^ The ongoing pandemic is a significant stressor and likely to worsen the already high prevalence of mental health concerns among PLWH.^45^

### Country level challenges

The rapidly growing pandemic has strained national healthcare systems worldwide. In Eastern Europe, HIV physicians have been called to care for patients with COVID-19, creating staffing shortages.^39^ This has forced some clinics to suspend in-person visits, others to postpone blood tests, and some to solely focus on ART distribution. Simultaneously, these already overwhelmed clinics are approached by PLWH who previously received their healthcare elsewhere but were unable to return home due to travel restrictions.

These challenges limit the ability of clinics to identify individuals with drug resistance, rapidly diagnose opportunistic infections, and carry out routine blood tests. For high risk individuals who wish to be tested for HIV, this delays linkage to care, making it impossible to “test and treat” in a timely manner. Modelling studies show that even a three-month disruption of ART supply can lead to half a million excessive HIV/AIDS related deaths in Africa alone, erasing years of progress.^46^

The challenges to HIV care mentioned above are simultaneously impacting TB care. TB is the leading cause of mortality among PLWH and modelling studies show that even short-term disruptions in TB diagnosis and treatment can lead to increased incidence and mortality for many years to come.^47^ One of the major strategies to tackle the spread of COVID-19 has been encouraging ‘shelter in place’ or home quarantine; prolonged contact at the household level can lead to household transmission of TB, whose long incubation period means we may not see the impact of this on TB incidence for months or years.^48^

Finally, an increased emphasis on COVID-19 should not distract from timely administration of annual influenza and guideline-directed pneumococcal vaccines to PLWH.

### Solutions

Despite such monumental challenges, this pandemic creates the opportunity to accelerate much needed changes in care delivery. Multi-month prescribing has now taken off in several African and Eastern European countries.^39^^,^^49^ While this has the obvious drawback of limited viral load/CD4 monitoring, it provides an opportunity for innovation in point-of-care testing solutions.

Moving forward, countries should work towards maintaining a steady supply of ARVs and anti-TB drugs, especially when exclusively imported. Notably, South Africa did not expand multi-month prescribing due to fears of ART shortages. This is necessary to buffer health systems against external shocks, especially in high HIV/TB prevalence settings.

Finally, solutions must be tailored in a location-specific manner-telemedicine visits help maintain consistent patient access to their providers in high resource settings.^50^ In low resource settings, decentralizing HIV testing will help both increase access to care and offload larger hospitals, which can focus on sicker patients. In Spain, some non-governmental organizations are involved in collecting medications for individuals and delivering them to their homes, which can be particularly effective for elderly individuals who are already at high risk of contracting COVID-19.^41^

The rapid spread of SARS-CoV-2 globally has created unprecedented challenges to health systems worldwide, making it inevitable that the world will miss the 2020 goal of fewer than 500,000 annual HIV/AIDS related deaths, but a loss of focus on HIV/AIDS programs amidst the global pandemic risks erasing years of progress.

## Conclusions

With over 24 million cases of COVID-19 worldwide and with numbers growing rapidly, it is important to focus on prevention strategies to mitigate the spread of COVID-19, especially in high HIV prevalence countries. The clinical course of COVID-19 among PLWH does not seem to be different than it is in the general population, but areas of concern include high inflammatory states from both conditions, which could result in complications. One lesson regarding therapeutics that can be borrowed from the HIV epidemic is that the first randomized control trial on zidovudine showed only a modest effect, but building on the results of that trial over years has led to the now ubiquitous highly effective ART. Similarly, new trials on COVID-19 therapeutics need to be built on the modest but promising results of remdesivir, with other agents augmenting its effect through complementary mechanisms. The global focus on the COVID-19 pandemic, including the race for a viable and effective vaccine and research into treatment options have the potential to synergistically drive progress for research in HIV infection.

## Funding

This research did not receive any specific grant from funding agencies in the public, commercial, or not-for-profit sectors.
